# A functional approach reveals cross-system bottom-up control on coral reefs

**DOI:** 10.1371/journal.pbio.3003251

**Published:** 2025-07-09

**Authors:** Sterling B. Tebbett, Scott D. Ling

**Affiliations:** 1 Institute for Marine and Antarctic Studies, University of Tasmania, Hobart, Australia; 2 College of Science and Engineering, James Cook University, Townsville, Queensland, Australia

## Abstract

Understanding how cross-system subsidies shape the functioning of recipient ecosystems across trophic levels was unresolved. This Primer explores a new study in PLOS Biology which reveals that seabird-derived nutrients fuel primary producers on coral reefs, enhancing herbivore productivity.

## Teaser

Understanding how cross-system subsidies shape the functioning of recipient ecosystems across trophic levels was unresolved. A new study in PLOS Biology reveals seabird-derived nutrients fuel primary producers on coral reefs, enhancing herbivore productivity.

**Fig 1:**
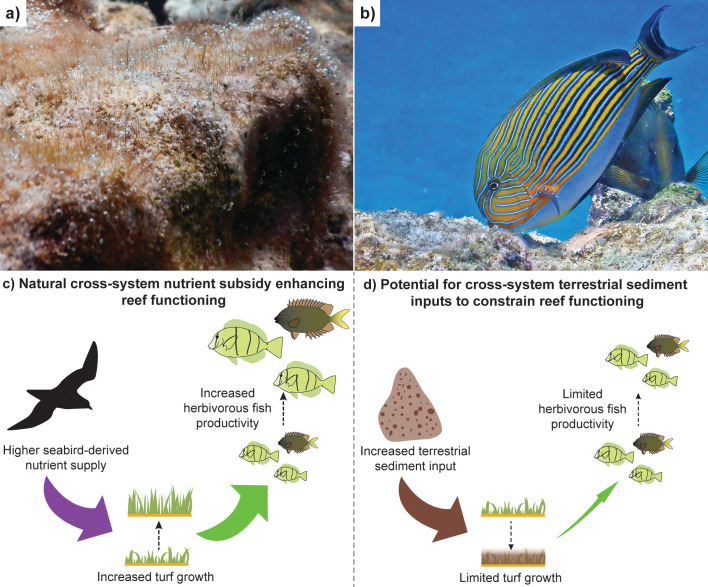
Cross-system connections can mediate the functioning of coral reefs via bottom-up control of productivity flowing through herbivorous trophic pathways. **(a)** Highly productive turf communities represent major primary producers on coral reefs (photograph: SB Tebbett). **(b)** Herbivorous fishes such as the turf cropping surgeonfish, *Acanthurus lineatus*, rely on the primary production of turfs to meet their nutritional needs (photograph: SB Tebbett). **(c)** A conceptual overview of the results of a new study in PLOS Biology by Benkwitt and colleagues [2] which quantified key ecosystem functions and demonstrated that seabird-derived nutrient supply can underpin strong cross-system connections between island ecosystems and adjacent coral reefs. Benkwitt and colleagues revealed that higher inputs of seabird nutrients fueled turf growth and indirectly enhanced the productivity of herbivorous fishes. **(d)** An alternative scenario where terrestrial sediment inputs underpin strong cross-system connections. However, in this case, sediments can limit turf growth and constrain herbivorous fish productivity [1,9]. These different scenarios underscore the importance of using a functional approach to quantify the strength of cross-system connections if we are to comprehensively understand how changing environmental conditions can have flow-on consequences for recipient ecosystems.

Aquatic and terrestrial ecosystems can be closely connected through the cross-system flux of energy and material [1]. Yet, ecosystems are often studied independently, stymieing our understanding of how changes in one system can have flow-on consequences for the other in a rapidly changing world [1]. Understanding these cross-system links can be a complex task given that the magnitude of energy and material fluxes are context-dependent, with asymmetries in the magnitude of inputs from different ecosystem components [1]. The complexity of understanding and tracing cross-system linkages is further magnified by the fact that ecosystems are intrinsically structured by both top-down and bottom-up forces, the relative strength of which can also vary tremendously under different contexts. While it is widely accepted that both top-down and bottom-up forces can shape the functioning of ecosystems (i.e., the rates at which energy or material are moved or stored in ecosystems), the ‘static’ metrics (e.g., the abundance of organisms) frequently used to quantify ecosystems can only provide partial insights into functioning [2]. By focusing on process-based metrics, a new study published in PLOS Biology by Benkwitt and colleagues [3] successfully links human-mediated changes on terrestrial island ecosystems to the functioning of adjacent coral reefs. The study reveals that the loss of seabirds on islands, due to invasive rats, reduces key natural nutrient subsidies to adjacent reefs, constraining the flow of productivity through primary producers to herbivorous fishes (Fig 1). These results underscore the potential for herbivorous trophic pathways on reefs to be mediated by bottom-up controls, with the process-based cross-system evaluation revealing the relative role nutrient subsidies play in this bottom-up control.

The rate at which energy flows through primary producers to herbivores is fundamental to the productivity of many ecosystems and has been a long-standing focus of ecological research [4,5]. In coral reef systems, herbivory is generally dominated by fishes, with coral reef fishes representing one of the most diverse, abundant, and widespread groups of vertebrate herbivores in the world [6] (Fig 1b). There have been substantial advances in our understanding of how herbivorous fishes influence reefs from the top-down (i.e., how herbivores shape communities of primary producers), just as there have been for herbivores in other ecosystems [7]. However, the understanding of bottom-up controls on herbivores in reef ecosystems has lagged, especially in respect to the magnitude of these bottom-up forces in different contexts. This lag in understanding is partly because, unlike plants on land, the predominant primary producers on coral reefs are small, hard to quantify, and often go overlooked.

On reefs, the primary productivity that fuels the herbivore trophic pathway is underpinned by ‘turfs’ [5] (Fig 1a). Turfs are communities of short (<2 cm) multi-species assemblages of algae and cyanobacteria that cover most hard reef surfaces that are not covered by corals [8]. The importance of understanding the role of these turfs has grown over recent years following the global decline in coral cover. As corals are lost, they are frequently replaced by turfs, with turf cover rather than coral cover often dominating on reefs [8]. The importance of turfs in reef primary productivity has long been recognized [5]. However, research into how turf productivity mediates herbivore communities via bottom-up control has been surprisingly limited—perhaps because of the diminutive size of turfs and the logistical difficulties associated with measuring turf growth and productivity. However, Benkwitt and colleagues [3] recognized the importance of examining turfs in this context to track how nutrient subsidies could have flow-on consequences for reef productivity. By estimating the magnitude of seabird-derived nitrogen inputs as well as measuring turfs at the scale of millimeters and examining their nutritional quality, Benkwitt and colleagues revealed that turfs grew faster and were more nutritious where seabird-derived nutrient subsidies were higher. These turf measurements were combined with static (e.g., biomass) and process-based (e.g., productivity) metrics of the herbivore community via a structural causal modeling approach. In doing so, it was found that the rate of nutritional resource production (i.e., turf growth), rather than nutritional quality or turf cover, related to the biomass and productivity of the herbivorous fish community (Fig 1c).

The findings of this new study in PLOS Biology emphasize the importance of a process-based approach to the quantification of ecosystems, as well as the need to comprehensively consider the context of the system in question to understand change. A focus on quantifying dynamic ecosystem processes invariably provides more direct information on the rate at which material or energy is cycling through an ecosystem compared to static measurements [2]. Despite often being harder to quantify, an enhanced focus on process-based metrics will yield more informative insights into how environmental change shapes the functioning of ecosystems, and ultimately an ecosystem’s capacity to support key services.

The finding that cross-system connections can significantly shape a core pathway of productivity on coral reefs also emphasizes the need to understand and quantify the magnitude of such cross-system connections more broadly under different contexts [1]. Cross-system connections are widespread between aquatic and terrestrial environments [1], and in a changing world these connections can be eroded, disrupting the functioning of ecosystems [3]. For example, outside of the marine realm, freshwater streams and adjacent terrestrial riparian ecosystems are also closely linked in a reciprocal nature [1]. Tree leaves that are deposited in streams represent a fundamental nutritional subsidy that underpins the productivity of aquatic detrital food webs [1]. Indeed, the larvae of various insects rely on these detrital resources in streams [1]. The productivity of this detrital pathway can also feedback into adjacent terrestrial ecosystems as insect larvae develop and emerge from the stream, whereby they become prey to terrestrial predators such as birds and reptiles [1]. Given that this reciprocal feedback is underpinned by the deposition of tree leaves, human actions that remove the trees will fundamentally disrupt and weaken these intricate reciprocal cross-system connections, likely eroding the productivity of both aquatic and terrestrial ecosystems.

Human actions can not only weaken cross-system connections, but they can also strengthen connections, disrupting recipient ecosystem functioning in a major way. For example, the flux of terrestrial sediments to aquatic ecosystems can be strengthened by human actions [9]. In respect to coral reefs, such increased terrestrial sediment inputs could represent the largest cross-system impact constraining the productivity of herbivorous pathways. A substantial proportion of the world’s coral reefs have been, and continue to be, impacted by higher sediment inputs due to land modifications such as deforestation [9]. These sediments can become trapped within the complex structure of turfs, with higher sediment loads strongly constraining turf growth and productivity yields to herbivorous fishes [2] (Fig 1d). As such, understanding how strengthening of land-sea connections via sediment inputs can reduce the productivity of herbivorous pathways represents a key area for ongoing research. This is particularly important given such reductions in productivity can have consequences for people via reduced fisheries yields [9].

Beyond coral reefs, the rise of sediment-laden turfs is also playing out across the world’s coastal temperate reef systems [10]. Understanding how increased sediment inputs are shaping the productivity of the world’s coastal reefs, in general, represents a tantalizing research question. However, given the scale, the diversity of the different terrestrial and reef ecosystems involved, the variety of bottom-up and top-down forces operating, and the inherent variability in the magnitude of sediment fluxes, this represents a challenging question to address. As Benkwitt and colleagues [3] demonstrate, it is possible to trace the magnitude of cross-system connections. Perhaps their study can act as a blueprint that can be scaled up to explore how other cross-system connections are reshaping the productivity of the world’s reef systems at global scales.
